# Genetic Characterization of Antibiotic Resistant *Enterobacteriaceae* Isolates From Bovine Animals and the Environment in Nigeria

**DOI:** 10.3389/fmicb.2022.793541

**Published:** 2022-02-25

**Authors:** Christiana Jesumirhewe, Burkhard Springer, Franz Allerberger, Werner Ruppitsch

**Affiliations:** ^1^Department of Pharmaceutical Microbiology, Prof Dora Akunyili College of Pharmacy, Igbinedion University, Okada, Nigeria; ^2^Institute of Medical Microbiology and Hygiene, Austrian Agency for Health and Food Safety, Vienna, Austria

**Keywords:** whole genome sequencing, *Enterobacteriaceae*, antibiotic resistance, bovine animals, environment

## Abstract

There is a link between antibiotic resistance in humans, livestock and the environment. This study was carried out to characterize antibiotic resistant bovine and environmental *Enterobacteriaceae* isolates from Edo state, Nigeria. A total of 109 consecutive isolates of *Enterobacteriaceae* were isolated from March–May 2015 from 150 fecal samples of healthy bovine animals from three farms at slaughter in Edo state Nigeria. Similarly, 43 *Enterobacteriaceae* isolates were also obtained from a total of 100 environmental samples from different sources. Isolates were recovered and identified from samples using standard microbiological techniques. Recovered isolates were pre-identified by the Microbact Gram-Negative identification system and confirmed with Matrix-assisted laser desorption ionization-time of flight (MALDI-TOF) mass spectrometry and ribosomal multilocus sequence typing (rMLST). Antibiotic susceptibility testing was carried out by Kirby-Bauer method for 14 antibiotics. Whole genome sequencing (WGS) was carried out for isolate characterization and identification of resistance determinants. Out of 109 animal and 43 environmental *Enterobacteriaceae* isolates, 18 (17%) and 8 (19%) isolates based on selection criteria showed antibiotic resistance and were further investigated by whole genome sequencing (WGS). Resistance genes were detected in all (100%) of the resistant bovine and environmental *Enterobacteriaceae* isolates. The resistance determinants included β-lactamase genes, aminoglycoside modifying enzymes, *qnr* genes, sulfonamide, tetracycline and trimethoprim resistance genes, respectively. Out of the 18 and 8 resistant animal and environmental isolates 3 (17%) and 2 (25%) were multidrug resistant (MDR) and had resistance determinants which included efflux genes, regulatory systems modulating antibiotic efflux and antibiotic target alteration genes. Our study shows the dissemination of antibiotic resistance especially MDR strains among Nigerian bovine and environmental *Enterobacteriaceae* isolates. The presence of these resistant strains in animals and the environment constitute a serious health concern indicated by the difficult treatment options of the infections caused by these organisms. To the best of our knowledge we report the first detailed genomic characterization of antibiotic resistance in bovine and environmental *Enterobacteriaceae* isolates for Nigeria.

## Introduction

Members of the family *Enterobacteriaceae* are important pathogens of humans and animals causing various infections for example in humans which include septicemia, pneumonia, peritonitis etc. (Makharita et al., 2020). They have been reported to be the main causes of nosocomial infections (Centers for Disease Control and Prevention [CDC], 2013). In animals, members of the family *Enterobacteriaceae* can be divided into three groups depending on their pathogenicity. They include major animal pathogens like *Escherichia coli*, opportunistic pathogens that occasionally cause infections in animals like *Proteus* spp., *Enterobacter* spp., *Citrobacter* spp. and organisms of uncertain importance for animals, e.g., *Erwina* spp., *Leclercia adecarboxylata* (Veterian Key, 2016). Several virulence factors involved in disease occurrence in members of the *Enterobacteriaceae* family have been previously studied which include invasion, hemolysins, siderophores and toxins which are controlled by specific virulence genes (Pearce et al., 2006).

Antibiotic resistance is a serious health concern not only among human pathogens but also in isolates found in other habitats. Many resistant pathogenic bacteria and commensals are found in different hosts, or in the environment (Taylor et al., 2001). The connections between humans, animals, and their environment allow the transfer of bacteria and mobile genetic elements between different compartments (Woolhouse and Ward, 2013). Antibiotic resistance genes are emerging environmental contaminants, as they can be transferred to other bacteria present in their environment (Krishnasamy, 2018). Multidrug resistance has increased all over the world which is considered a public health threat. Several recent investigations have reported the emergence of multidrug resistant bacterial pathogen from different origins including humans, birds, cattle, and fish (Algammal et al., 2020, 2021a,b). This increases the need for routine application of antimicrobial susceptibility testing to detect the antibiotic of choice as well as screening of the emerging MDR strains. There are different mechanisms of resistance in members of the family *Enterobacteriaceae*. They include modification of the target, efflux phenomenon, impermeability, and enzymatic inactivation (Kotsyuba et al., 2014). Understanding resistance mechanisms in *Enterobacteriaceae* including how they develop and are transmitted is important for developing ways to monitor resistance spread effectively and also have effective treatment options. The various resistance determinants can be exploited to produce possible safe and potent novel antimicrobial drugs with new mechanisms of action.

Livestock animals are linked to humans through the food chain and the environment they share (Marshall and Levy, 2011). Over the years, antimicrobials have been applied for treatment of diseases in food producing animals, but also for growth promotion and prophylactic purposes. Multiple countries including developed and developing nations like Nigeria have introduced bans on the non-therapeutic use of antimicrobials. However, the coordinated surveillance and monitoring of antimicrobial use and resistance especially in developing countries like Nigeria is still limited, resulting in an increase of resistant pathogens. Most of the main classes of antibiotics used in human medicine are represented in a list of antibiotics tagged as “critically important” for livestock animals by the World Organization for Animal Health (OIE, 2007). Antibiotic residues are released into the environment as a result of inadequate absorption and metabolic activities carried out by food animals. Antimicrobials added to food for livestock animals can be disseminated as a result of leaching and release by urine and feces (Krishnasamy, 2018). The presence of antibiotic resistance in *Enterobacteriaceae* as well as other bacteria has been well documented in human, animal and environmental isolates in developed countries (Yates et al., 2004; Hoyle et al., 2005; Aarestrup et al., 2009; Fischer et al., 2012; Spoor et al., 2012). In low/middle income countries like Nigeria, research focus is majorly on antimicrobial resistance in human pathogens with only a few detailed reports on animal and environmental isolates (Adelowo and Fagade, 2009; Olowe et al., 2015; Ayeni et al., 2016; Ngbede et al., 2017; Adelowo et al., 2018; Shivakumaraswamy et al., 2019). The aim of this study was to investigate antibiotic resistance and characterize the resistance mechanism in *Enterobacteriaceae* isolated from bovine animals and the environment in Edo state Nigeria using whole genome sequencing.

## Materials and Methods

### Bacterial Isolation and Identification

A total of 109 consecutive isolates of *Enterobacteriaceae* were isolated from March–May 2015 from 150 fecal samples of healthy bovine animals at slaughter from three farms in Edo state Nigeria. The animals at slaughter were mostly Nigerian native cow breeds of both male and female sex. Further, 43 *Enterobacteriaceae* isolates were obtained from 100 environmental samples collected at six different sites in Edo state, Nigeria during the same period: 50 samples from refuse dump sites, 20 samples from flowing rivers, 20 samples from the soil and 10 samples from waste water. The environmental sampling sites were in close proximity with the animal farms. Isolates were identified using standard microbiological techniques (Cheesebrough, 2006). For the isolation and identification of the different members of *Enterobacteriaceae*, samples were inoculated on MacConkey agar plates (Oxoid, Hampshire, United Kingdom) and incubated for 24 h at 37°C. Distinct colonies were obtained from the agar plates and subcultured to obtain pure colonies. The Microbact Gram-Negative identification system (Oxoid, Basingstoke Hampshire, United Kingdom) was used in the preliminary identification of aerobic and facultative anaerobic Gram-negative bacteria (*Enterobacteriaceae*) (Mugg and Hill, 1981; Thomas, 1983). Organisms were identified based on pH change and substrate utilizations as established by previous reference methodologies (Cowan and Steel, 1977; Farmer 3^rd^ et al., 1985; Balows et al., 1991). Preidentified isolates were further subcultured on Drigalski Lactose agar (Oxoid, Hampshire, United Kingdom) and subsequently confirmed by MALDI-TOF mass spectrometry (Bruker Daltonik GmbH, Bremen, Germany) analysis.

### Antimicrobial Susceptibility Testing

The Kirby-Bauer susceptibility testing technique (Bauer et al., 1966) was performed and results were interpreted using European Committee on Antimicrobial Susceptibility Testing (EUCAST) criteria (European Committee on Antimicrobial Susceptibility Testing [EUCAST], 2015). The isolates were tested based on selection criteria with 14 antibiotics belonging to 11 classes of antimicrobial agents: carbapenems; meropenem (10 μg), ertapenem (10 μg), extended spectrum cephalosporins; ceftazidime (10 μg), cefotaxime (5 μg), cefepime (30 μg), penicillin + β-lactamase inhibitor; amoxicillin/clavulanic acid (30 μg), cephamycin; cefoxitin (30 μg), monobactam; aztreonam (30 μg), folate pathway inhibitors; trimethoprim (5 μg), fluoroquinolones; ciprofloxacin (5 μg), levofloxacin (5 μg), aminoglycosides; amikacin (30 μg), antipseudomonal penicillins + β-lactamase inhibitor; piperacillin/tazobactam (36 μg), phenicols; chloramphenicol (30 μg) (Oxoid, Basingstoke Hampshire, United Kingdom) on Mueller Hinton agar (Oxoid, Hampshire, United Kingdom) plates. These antibiotics are clinically important drugs both in the human and veterinary health sectors in Nigeria. Multidrug resistant isolates were identified among the tested isolates. Multidrug resistance was defined as non-susceptibility of an *Enterobacteriaceae* isolate to ≥1 agent of ≥3 antimicrobial classes (Magiorakos et al., 2012).

### Whole Genome Sequencing

Whole genome sequencing (WGS) was carried out for 18 resistant/multidrug-resistant bovine animal isolates and 8 resistant/multidrug-resistant environmental isolates based on selection criteria. The MagAttract HMW DNA extraction kit (Qiagen, Hilden, Germany) was used for the extraction of genomic DNA (gDNA). Quantification of genomic DNA was carried out on a Qubit™ 2.0 Fluorometer using the dsDNA BR Assay kit (Invitrogen by Thermo Fisher Scientific, Waltham, MA, United States). Preparation of fragment libraries of the bacterial genomes was carried out using the Illumina Nextera XT DNA library preparation kit (Illumina Inc., San Diego, CA, United States). A paired end sequencing using a read length of 2 × 300 bp on an Illumina Miseq (Miseq v3.0, Illumina Inc., San Diego, CA, United States) was performed using Miseq reagent kit v3 containing the reagent cartridge and flow cell.

Raw reads (FASTQ files) were trimmed at their 5′ and 3′ ends until an average base quality of 30 was reached in a window of 20 bases, and Velvet version 1.1.04 (Zerbino, 2010) was used in carrying out the assembly using optimized k-mer size and coverage cutoff values based on the average contigs length with >1,000 bp. Species identification via MALDI-TOF MS was confirmed using ribosomal multilocus sequence typing (rMLST)^1^. Assembled genomes were uploaded to the ResFinder 2.1 web server^2^ (Zankari et al., 2012) and to the Comprehensive Antibiotic Resistance Database-Resistance Gene Identifier (CARD-RGI) (Jia et al., 2017) to identify antimicrobial resistance genes. ARGs were identified based on a minimum cutoff of 98% nucleotide identity for perfect or strict hits predicted by RGI. The Enterobase core genome multilocus sequence typing (cgMLST) scheme comprising 2,513 core genes^3^ was used for strain comparison using SeqSphere + version 7.7.5 (Ridom, Münster, Germany) as described recently (Bernreiter-Hofer et al., 2021; Cabal et al., 2021; Mišić et al., 2021). Multilocus sequence typing [MLST; MLST 1.8, Centre for Genomic Epidemiology (CGE), Lyngby DK] was used to type resistant isolates. *Klebsiella variicola* isolates were typed also by multilocus sequence typing [MLST *Klebsiella variicola*, Instituto Nacional de Salud Pública (INSP)^4^] (Barrios-Camacho et al., 2019). Plasmids on the draft genomes of the resistant *Enterobacteriaceae* isolates were analyzed and classified using PlasmidFinder 1.3 webtool^5^ based on a threshold of 95% ID (Carattoli et al., 2014). Plasmid replicons and Inc plasmid groups were identified.

### Genomes From Sequence Read Archive

Three genomes of isolates in this study were downloaded from the Sequence Read Archive (SRA) Bioproject PRJEB20802 (Ayandiran et al., 2018).

### Phylogenetic Analysis

Whole genome based cgMLST phylogenetic analysis including three genomes from Nigerian *Escherichia coli* poultry isolates retrieved from SRA with accession ERR1986373–ERR1986375 and all bovine *Escherichia coli* isolates (*n* = 12) in this study was carried out and a minimum spanning tree was calculated. The 15 isolates had eight sequence types (ST). In this study, Multilocus sequence typing (MLST) revealed seven STs (ST46, ST111, ST23, ST155, ST10, ST58, ST423) for *Escherichia coli* (*n* = 12) animal isolates (Supplementary Table 1).

### Nucleotide Sequence Accession Numbers

This Whole Genome Shotgun project has been deposited at DDBJ/ENA/GenBank under the accession numbers JAIKTX000000000-JAIKUW000000000. The version described in this paper is version JAIKTX010000000-JAIKUW010000000.

### Statistical Analyses

The correlation analyses between the tested antimicrobial agents as well as the phenotypic resistance pattern and the detected resistance genes were performed using SPSS statistical software package version 20.0 (IBM SPSS Inc., New York, NY, United States).

## Results

### Phenotypic Characteristics of *Enterobacteriaceae* in the Samples

The recovered *Enterobacteriaceae* isolates were pink (lactose fermenters) and colorless (non- lactose fermenters) colonies on MacConkey agar. Preliminary biochemical tests on the recovered isolates using the Microbact Gram-Negative identification system gave varying reactions to the different tests carried out. Biochemical tests included lysine decarboxylase, ornithine decarboxylase, H_2_S, Glucose, Mannitol, Xylose, ONPG, Indole, Urease, VP, Citrate and TDA. On Drigalski agar, *Enterobacteriaceae* isolates were yellow colonies (*E. coli, Klebsiella, Enterobacter* spp.) and blue-gray to blue-green colonies (*Proteus, Serratia, Providencia* spp.). Species identification using MALDI-TOF-MS assigned the 109 animal isolates to 9 species and the 43 environmental isolates to 10 species (Supplementary Tables 2, 3).

### Antibiogram and Phenotypic Resistance Pattern of the Isolates

Out of the 109 animal and 43 environmental *Enterobacteriaceae* isolates, 18(17%) and 8(19%) isolates revealed antibiotic resistance in the Kirby-Bauer susceptibility testing technique (Supplementary Tables 4a,b). Species identification using MALDI-TOF-MS and ribosomal MLST assigned the 18 resistant animal isolates to 5 species and the 8 resistant environmental isolates to 7 species (Supplementary Table 5). Resistant animal isolates included *Escherichia coli, Serratia marcescens, Klebsiella quasipneumoniae, Klebsiella variicola, Proteus terrae* while the resistant environmental isolates included *Enterobacter quasiroggenkampii, Enterobacter hormachei, Citrobacter koseri, Klebsiella quasipneumoniae, Klebsiella variicola, Proteus terrae, and Proteus faecis* (Supplementary Table 5). The selected animal strains were resistant to trimethoprim (61%), cefoxitin (50%), amoxicillin-clavulanic acid (50%), while environmental strains were resistant to trimethoprim (75%) and cefoxitin (50%) (Supplementary Table 6).

### Antimicrobial Resistance Genes of the Recovered Resistance Isolates

Whole genome sequencing revealed that all of the 18 resistant animal isolates harbored more than one resistance gene. Similarly, all the resistant environmental strains had multiple resistance genes detected by WGS.

**FIGURE 1:**
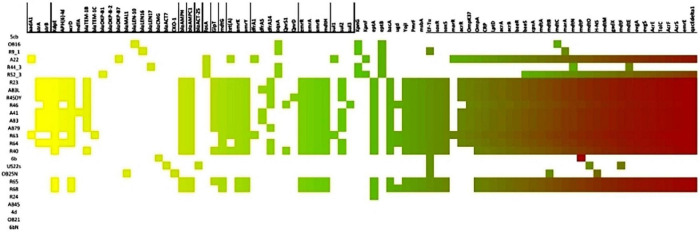
Heat map showing the distribution of antimicrobial resistance genes among the recovered isolates.

The resistance determinants in the animal isolates included β-lactamase genes, *bla*_OKP–B–1_, *bla*_OKP–B–7_, *bla*_TEM–1b_, *bla*_TEM–1c_, *bla*_LEN–16_, *bla*_LEN–17_, *bla*_AMPC1_, *bla*_AMPH_; streptomycin resistance genes, *str*A and *str*B; aminoglycoside modifying enzymes, *aad*A1; fosfomycin resistance determinant, *fos*A, *glp*T; *qnr* genes, *qnr*D, *qnr*S1 and plasmid encoded efflux pump, *oqx*A; sulfonamide resistance genes, *sul*1, *sul*2, and *sul*3; tetracycline resistance gene, *tet*(A) and trimethoprim resistance genes, *dfr*A1, *dfr*A5, *dfr*A14. The resistance determinants in the environmental isolates included β-lactamase genes*: bla*_OKP–B–2_, *bla*_ACT–7_, *bla*_ACT–25_ *bla*_CMG_, *bla*_CKO–1_, *bla*_TEM–1b_, *bla*_MAL–1_, *bla*_LEN–10_, Sulfonamide resistance gene, *sul*2, phenicol resistance gene, *cat*II, tetracycline resistance genes, *tet*(A), *tet*(D) and trimethoprim resistance gene, *dfr*A14. Other resistance determinants which included efflux genes *emr*A, *emr*B, *emr*K, *emr*Y, *msb*A, regulatory systems modulating antibiotic efflux H-NS, *mar*R, *emr*R, *mar*A, CRP, antibiotic target alteration gene *bac*A, *Pmr*F, *ept*A, EF-Tu mutants, ugd, were also detected in the antibiotic resistant animal and environmental isolates. Supplementary Table 7 shows the characteristics of the antibiotic resistant animal and environmental isolates. Figure 1 illustrates the distribution of the antimicrobial resistance genes among the recovered isolates. Ten plasmid incompatibility groups were identified among the animal *Enterobacteriaceae* isolates with IncF family types being predominant. Four plasmid incompatibility groups were identified among the antibiotic resistant environmental *Enterobacteriaceae* isolates with IncF family types also being predominant (Supplementary Table 1).

### Correlation Between the Phenotypic and Genotypic Multidrug-Resistance Patterns

The antibiogram of the resistant recovered animal isolates showed that one isolate (6%) is multidrug-resistant (MDR) (MDR: non-susceptibility of the isolate to ≥1 agent of ≥3 antimicrobial classes) to cephalosporins: ceftazidime, cefotaxime, cephamycins: cefoxitin, monobactams: aztreonam, aminoglycosides: amikacin, antipseudomonal penicillins + β-lactamase inhibitor: piperacillin/tazobactam, folate pathway inhibitors: trimethoprim and had the regulatory system modulating antibiotic efflux CRP as resistance determinant. Out of the resistant animal isolates, two isolates (11%) were multidrug-resistant (MDR) to cephamycins: cefoxitin, folate pathway inhibitors: trimethoprim, penicillin + β-lactamase inhibitor: amoxicillin/clavulanic acid and had varying resistance determinants including regulatory systems modulating antibiotic efflux: CRP, H-NS, *mar*R, *emr*R, *mar*A, efflux genes: *emr*A, *emr*B, *emr*K, *emr*Y, antibiotic target alteration genes: *bac*A, pmrF, *ept*A, EF-Tu mutants, beta-lactamase resistance genes: *bla*_AMPC_, *bla*_AMPH_, *bla*_TEM1_, sulfonamide resistant gene *sul*2, quinolone resistance gene *qnr*S1, trimethoprim resistant dihydrofolate reductase *dfr*A14 etc.

One of the resistant environmental isolates (13%) was multidrug-resistant to cephalosporins: ceftazidime, cefotaxime, cefepime, cephamycins: cefoxitin, monobactams: aztreonam, aminoglycosides: amikacin, antipseudomonal penicillins + β-lactamase inhibitor: piperacillin/tazobactam, folate pathway inhibitors: trimethoprim and had the regulatory system modulating antibiotic efflux CRP, beta-lactamase resistance genes: *bla*_MAL1_, *bla*_CKO–1_, elfamycin resistance: EF-Tu mutation as its resistance determinants while another isolate (13%) was multidrug resistant to cephalosporins: ceftazidime, cefotaxime, monobactams: aztreonam, aminoglycosides: amikacin, folate pathway inhibitors: trimethoprim and had the regulatory system modulating antibiotic efflux CRP as resistance determinant (Supplementary Table 8).

The correlation coefficient estimated between different tested antimicrobial agents as well as the phenotypic resistance pattern and the detected resistance genes showed a positive correlation, i.e., the correlation is statistically significant as calculated p-value is less than 0.05.

### Genetic Comparison of Bovine *Escherichia coli* Isolates

**FIGURE 2:**
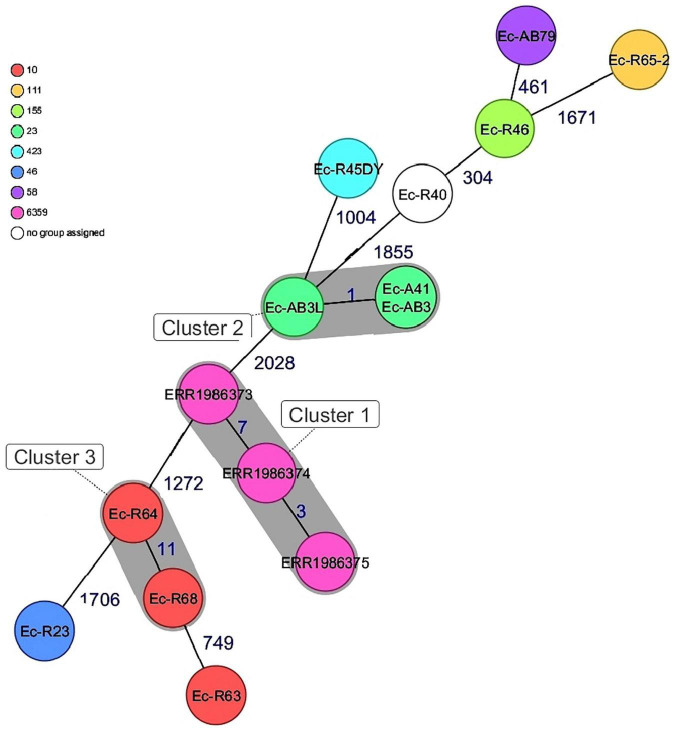
Minimum spanning tree for 15 *E. coli* isolates based on cgMLST of *E.coli*. Colors corresponds to the sequence types of the isolates. Each circle represents isolates with an allelic profile based on the sequences of 2520 core genome targets. Isolates with closely related genotypes were identified with a maximum of 11 allelic differences and are shaded in gray.

CgMLST of the 15 isolates showed an allelic distance from one to 2028. Bovine *Escherichia coli* isolates in this study differed among each other in a minimum of one and a maximum of 1855 alleles. Based on the defined complex threshold (CT) of 20 allelic differences (Leopold et al., 2014) three clusters were obtained. The three Nigerian poultry isolates were closely related with a maximum allelic difference of 7 and were all located in cluster 1 and differed by a minimum of 1272 alleles from the bovine isolates from this study. Three bovine isolates (cluster 2, ST23) obtained from two different farms that were not in close proximity were closely related with a maximum allelic difference of 1. Two other bovine isolates (ST10) in this study from the same farm were also closely related with an allelic difference of 11 (Figure 2). The poultry isolates used in the strain comparison were obtained from a different state far from the farm locations in this study hence no close relatedness was observed.

### Multilocus Sequence Typing of Other Recovered Isolates

The animal *Klebsiella variicola* isolates had ST227 and 259 respectively. One animal *Klebsiella quasipneumoniae* had ST1136. The *Proteus terrae, Serratia marcescens*, and one *Klebsiella quasipneumoniae* animal isolate had previously unknown sequence types. One resistant *E. coli* animal isolate also had a new sequence type. The resistant *Proteus terrae, Proteus faecis, Citrobacter koseri, Enterobacter hormachei, Enterobacter quasiroggenkampii, Klebsiella quasipneumoniae* environmental isolates also had new sequence types (Supplementary Table 1). The *Klebsiella. variicola* environmental isolate was assigned to the new ST314.

## Discussion

*Enterobacteriaceae* are typically found in animals and the environment (Otokunefor et al., 2018) and have been reported as reservoirs of resistance genes that could be passed to other bacterial cells in the system. The use of antibiotics in livestock production results in an increase in antibiotic resistant strains in people (Bevan et al., 2017). Sewage, humans, companion, and domestic animals and the industries have been previously reported as sources of resistant microorganisms to the environment in African settings (Moremi et al., 2017). Determination of antimicrobial resistance in bacteria using phenotypic characterization is very important for therapeutic purposes, but further genetic characterization may sometimes seems appropriate. Whole-genome sequencing plays a key role in enhancing our understanding of how bacteria evolve, are transmitted, and monitoring of antimicrobial resistance. Regardless of the source of isolation in this study, the phenotypic antimicrobial susceptibility testing revealed high frequency of resistance to trimethoprim and cefoxitin. Recently, plasmid mediated FOX-1 AmpC ß-lactamases were detected in *Escherichia coli* isolates from cows in Nigeria (Ejikeugwu et al., 2018). A recent review on antimicrobial use revealed a high level of antimicrobial usage of tetracyclines, aminoglycosides and penicillins in animal production systems in Africa because they belong to the cheapest antibiotics (Kimera et al., 2020). The previous report showed high prevalence of antimicrobial resistance including multidrug resistance in the environment. Reports from developing countries especially in Africa are of major public health concern as only limited diagnostic and therapeutic options are available (Kimera et al., 2020). The presence of antimicrobial-resistant (AMR) and multidrug- resistant (MDR) bacteria that colonize the gut of animals might play an important epidemiological role in the spread of antimicrobial resistance between livestock animals and humans, either directly or through consumption of contaminated food. Several mechanisms of bacterial resistance to antimicrobial agents have been previously reported (Kumar and Varela, 2013; Sun et al., 2014). They include antibiotic efflux, antibiotic target alteration, antibiotic inactivation, antibiotic target replacement and antibiotic target protection. In this study various resistance determinants that encode antimicrobial efflux pumps were detected both in the resistant and multidrug resistant isolates. Over-expression of such determinants has been connected to a rise of clinically important multidrug-resistant pathogens (Nikaido, 1994). The regulatory system modulating antibiotic efflux (Resistance-Nodulation-Division multidrug efflux pumps) for example has been previously reported as an important determinant of multidrug resistance in Gram-negative bacteria (Fernando and Kumar, 2013). Resistance determinants that encode multidrug efflux pumps of the major facilitator superfamily were also detected in this study and have also been previously reported to contribute to the emergence of multidrug-resistant organisms (Kumar et al., 2020). Results of this study underline the wide distribution and importance of this resistance mechanism.

Some African studies identified antimicrobial resistance genes [*bla*_CTX–M_, *bla*_TEM_, *bla*_SHV_, *bla*_OXA_, *aac(6′)-lb-cr*, *tet(A), tet(B), sul1, sul2*, *qnr*] in bacteria from humans, animals, and environmental sources (Hamza et al., 2016; Ribeiro et al., 2016; Eguale et al., 2017; Seni et al., 2018), indicating that drug resistant pathogens have high tendency to spread widely (De Boeck et al., 2012; Ekwanzala et al., 2018; Oloso et al., 2018). Results from this study correlate with the previous African reports as *bla*_TEM_, *aph*(6)-*ld*, *tet(A), tet(D), sul1, sul2*, *qnr, dfr*A1, *dfr*A5, *dfr*A14 were some identified resistance genes in the resistant *Enterobacteriaceae* isolates. Significantly, no extended spectrum beta-lactamase (ESBL) gene was detected in the bovine and environmental *Enterobacteriaceae* isolates. This is in contrast with a recent report on human clinical *Enterobacteriaceae* isolates collected from the same geographical location that had a high rate of ESBL genes detected among the isolates (Jesumirhewe et al., 2020).

The role of *Enterobacteriaceae* as a reservoir for ESBL genes and other resistance determinants is enhanced by the presence of IncF plasmid family type which evolves quickly by replicon diversification and acquisition of antibiotic resistance traits (Carattoli, 2013). Various plasmid replicon types detected among the resistant isolates in this study indicate their importance for dissemination of antibiotic resistance. For example, in this study, resistance genes mediating quinolone resistance and transmitted by plasmids, *oqx*A, *qnr*D, and *qnr*S1 were detected in the resistant animal and environmental isolates indicating that resistance to quinolones was promoted through the plasmid-mediated determinant. Plasmid-mediated quinolone resistance (PMQR) determinants play an important role in the transmission of resistance among bacterial isolates (Santhosh et al., 2017). The predominating presence of IncF plasmid replicon type in the animal and environmental isolates increases the possibility of acquiring more resistance determinants as well as the emergence of novel resistance determinants (Amos et al., 2014). Efflux pumps have been previously reported to be encoded by plasmid borne genetic elements which plays an important role in the transmission of multidrug-resistance (Nikaido, 1994; Nelson and Levy, 2011).

Most developing countries in Africa like Nigeria have been reported to lack effective antimicrobial surveillance systems or are at different stages of developing them (Alonso et al., 2017; Kimera et al., 2020). Monitoring the emergence and spread of antibiotic resistant isolates may assist in developing strategies for treatment and prevention of infections especially for animals which have limited choice of prophylactic and therapeutic antimicrobials. A systematic antimicrobial resistance surveillance using more advanced techniques like WGS is required especially in African settings to detect new antibiotic resistance mechanisms and to determine the virulence of multidrug resistance pathogens for risk assessment and prevention of infection (Hamza et al., 2016; Grundmann and Gelband, 2018). Data from WGS could assist in planning effective interventional measures.

This study provides detailed genomic characterization of antibiotic resistance in bovine animal and environmental *Enterobacteriaceae* isolates for Nigeria. A number of previous Nigerian studies described the detection, frequency/prevalence of resistance to antibiotics in livestock and the environment using phenotypical techniques (Ayandiran et al., 2014; Onuoha, 2017; Otokunefor et al., 2018; Ugwu et al., 2018). Only a few Nigerian studies have been able to explore the genetic mechanisms of antibiotic resistance in bovine animals and the environment (Olowe et al., 2015; Sharma et al., 2017; Adelowo et al., 2018; Ayandiran et al., 2018; Ejikeugwu et al., 2018) which is important to understand the dissemination of resistant isolates. Further studies to characterize prevalent clones and plasmids that harbor antibiotic resistance genes are required.

Whole genome based cgMLST phylogenetic analysis of the bovine *Escherichia coli* isolates suggest a probable clonal spread of the isolates of different sequence types and plasmid replicon types. ST10 is an important multilocus sequence type detected in three animal *Escherichia coli* isolates in this study. Previously, ST10 *Escherichia coli* have been detected in chicken and have been reported as antibiotic resistant ESBL-producers (Chen et al., 2016). Previous reports show *Escherichia coli* ST10 to be commonly associated with other animals and humans (Ewers et al., 2012; Chen et al., 2016). This is consistent with our results as the isolates were found in feces of bovine animals. One limitation of this study is that samples obtained were not sufficient to get a large number of isolates to analyze possible links between the bovine and environmental isolates. Although resistance determinants were detected in both the animal and environmental isolates it would be important to further analyze isolates to characterize the possible linkages between animals and the environment.

## Conclusion

We report a wide dissemination of antibiotic resistant and multidrug resistant Nigerian bovine and environmental *Enterobacteriaceae* isolates. The emergence of these resistant strains is of public health concern indicated by the difficult treatment options in infections they cause. Very little data especially genotypic studies of isolates from animals and the environment exist for surveillance of antimicrobial resistance in most developing countries like Nigeria. It is necessary for developing countries like Nigeria to carry out surveillance systems involving a “One Health” approach which would be important to monitor transmission events. Support from global antimicrobial resistance networks should be sought to assist in developing and implementing antimicrobial resistance surveillance under one health approach.

## Data Availability Statement

The datasets presented in this study can be found in online repositories. The names of the repository/repositories and accession number(s) can be found in the article/Supplementary Material.

## Author Contributions

CJ conceived, designed, performed the experiments and wrote the manuscript. CJ, BS, FA, and WR analyzed the data. All the authors contributed to the article and approved the submitted version.

## Conflict of Interest

The authors declare that the research was conducted in the absence of any commercial or financial relationships that could be construed as a potential conflict of interest.

## Publisher’s Note

All claims expressed in this article are solely those of the authors and do not necessarily represent those of their affiliated organizations, or those of the publisher, the editors and the reviewers. Any product that may be evaluated in this article, or claim that may be made by its manufacturer, is not guaranteed or endorsed by the publisher.

## Footnotes

1
https://pubmlst.org/species-id

2
http://www.genomicepidemiology.org

3
https://enterobase.readthedocs.io/en/latest/pipelines/escherichia-statistics.html

4
https://mlstkv.insp.mx/

5
http://cge.cbs.dtu.dk/services/PlasmidFinder
