# Supplementary material for: Genetic Characterization of Antibiotic Resistant Enterobacteriaceae Isolates From Bovine Animals and the Environment in Nigeria
Source: Front Microbiol. 2022 Feb 25;13:793541. doi: 10.3389/fmicb.2022.793541 (PMC8916115; doi:10.3389/fmicb.2022.793541)
Supplement: Supplementary file 2 [file Table_2.docx]

| Number | Location | Identification | *Organism* | Source | Animal |
| --- | --- | --- | --- | --- | --- |
| 1 | Okada | R62 | *K. pneumoniae* | Feaces | Bovine |
| 2 | Okada | R97 | *C. freundii* | Feaces | Bovine |
| 3 | Okada | R51 | *K. pneumoniae* | Feaces | Bovine |
| 4 | Okada | R75 | *E. coli* | Feaces | Bovine |
| 5 | Okada | R41 | *E. coli* | Feaces | Bovine |
| 6 | Okada | R44E | *E. cloacae* | Feaces | Bovine |
| 7 | Okada | R72 | *E. asburiae* | Feaces | Bovine |
| 8 | Okada | R64 | *E. coli* | Feaces | Bovine |
| 9 | Okada | R69 | *E. coli* | Feaces | Bovine |
| 10 | Okada | R68 | *E. coli* | Feaces | Bovine |
| 11 | Okada | R60 | *E. coli* | Feaces | Bovine |
| 12 | Okada | R78 | *E. coli* | Feaces | Bovine |
| 13 | Okada | R71 | *E. cloacae* | Feaces | Bovine |
| 14 | Okada | R77E | *E. coli* | Feaces | Bovine |
| 15 | Okada | R101 | *S. marcescens* | Feaces | Bovine |
| 16 | Okada | R48 | *K. pneumoniae* | Feaces | Bovine |
| 17 | Okada | R65-2 | *E. coli* | Feaces | Bovine |
| 18 | Okada | R42 | *E. coli* | Feaces | Bovine |
| 19 | Okada | R52-2 | *E. asburiae* | Feaces | Bovine |
| 20 | Okada | R77 | *E. coli* | Feaces | Bovine |
| 21 | Okada | R90 | *E. coli* | Feaces | Bovine |
| 22 | Okada | R57 | *E. coli* | Feaces | Bovine |
| 23 | Okada | R87 | *E. coli* | Feaces | Bovine |
| 24 | Okada | R55 | *E. asburiae* | Feaces | Bovine |
| 25 | Okada | R63 | *E. coli* | Feaces | Bovine |
| 26 | Okada | R103 | *S. marcescens* | Feaces | Bovine |
| 27 | Okada | R73 | *E. asburiae* | Feaces | Bovine |
| 28 | Okada | R47 | *E. asburiae* | Feaces | Bovine |
| 29 | Benin | AB65-2 | *S. marcescens* | Feaces | Bovine |
| 30 | Okada | R46 | *E. coli* | Feaces | Bovine |
| 31 | Okada | R44-2 | *E. asburiae* | Feaces | Bovine |
| 32 | Okada | R43 | *E. cloacae* | Feaces | Bovine |
| 33 | Okada | R47E | *E. coli* | Feaces | Bovine |
| 34 | Okada | R102 | *E. asburiae* | Feaces | Bovine |
| 35 | Okada | R45-3 | *K. pneumoniae* | Feaces | Bovine |
| 36 | Okada | R40 | *C. freundii* | Feaces | Bovine |
| 37 | Okada | R49 | *E. coli* | Feaces | Bovine |
| 38 | Okada | R74 | *E. asburiae* | Feaces | Bovine |
| 39 | Okada | R100 | *E. coli* | Feaces | Bovine |
| 40 | Benin | AB79 | *E. coli* | Feaces | Bovine |
| 41 | Okada | R26 | *C. freundii* | Feaces | Bovine |
| 42 | Okada | R29 | *E. coli* | Feaces | Bovine |
| 43 | Benin | AB3 | *E. coli* | Feaces | Bovine |
| 44 | Okada | R14 | *K. pneumoniae* | Feaces | Bovine |
| 45 | Benin | AB15 | *E. coli* | Feaces | Bovine |
| 46 | Okada | R5 | *E. cloacae* | Feaces | Bovine |
| 47 | Benin | AB39L | *K. pneumoniae* | Feaces | Bovine |
| 48 | Okada | R35 | *K. pneumoniae* | Feaces | Bovine |
| 49 | Okada | A41 | *E. coli* | Feaces | Bovine |
| 50 | Okada | R36 | *E. coli* | Feaces | Bovine |
| 51 | Okada | A22 | *K. pneumoniae* | Feaces | Bovine |
| 52 | Okada | R33 | *E. coli* | Feaces | Bovine |
| 53 | Okada | A24 | *S. marcescens* | Feaces | Bovine |
| 54 | Benin | AB3L | *E. coli* | Feaces | Bovine |
| 55 | Okada | R13 | *K. pneumoniae* | Feaces | Bovine |
| 56 | Okada | R39 | *E. asburiae* | Feaces | Bovine |
| 57 | Okada | R27 | *C. freundii* | Feaces | Bovine |
| 58 | Okada | R2 | *K. pneumoniae* | Feaces | Bovine |
| 59 | Benin | AB8-2 | *K. pneumoniae* | Feaces | Bovine |
| 60 | Benin | AB55 | *P. vulgaris* | Feaces | Bovine |
| 61 | Okada | R18E2 | *C. freundii* | Feaces | Bovine |
| 62 | Okada | R25-1 | *E. coli* | Feaces | Bovine |
| 63 | Okada | R8 | *E. asburiae* | Feaces | Bovine |
| 64 | Okada | R24 | *K. pneumoniae* | Feaces | Bovine |
| 65 | Okada | A18 | *P. vulgaris* | Feaces | Bovine |
| 66 | Benin | AB45 | *P. penneri* | Feaces | Bovine |
| 67 | Benin | AB57 | *P. vulgaris* | Feaces | Bovine |
| 68 | Okada | A33 | *P. vulgaris* | Feaces | Bovine |
| 69 | Okada | R18E | *C. freundii* | Feaces | Bovine |
| 70 | Okada | R17 | *E. cloacae* | Feaces | Bovine |
| 71 | Okada | R45-2 | *E. asburiae* | Feaces | Bovine |
| 72 | Okada | R3 | *E. cloacae* | Feaces | Bovine |
| 73 | Okada | A40 | *P. vulgaris* | Feaces | Bovine |
| 74 | Benin | AB65-1 | *C. freundii* | Feaces | Bovine |
| 75 | Benin | AB29 | *E. coli* | Feaces | Bovine |
| 76 | Okada | R19 | *C. freundii* | Feaces | Bovine |
| 77 | Okada | R9-2 | *C. freundii* | Feaces | Bovine |
| 78 | Benin | AB9-2 | *E. cloacae* | Feaces | Bovine |
| 79 | Okada | R31 | *C. freundii* | Feaces | Bovine |
| 80 | Okada | R12-2 | *C. freundii* | Feaces | Bovine |
| 81 | Okada | R21 | *E. asburiae* | Feaces | Bovine |
| 82 | Okada | R22 | *E. asburiae* | Feaces | Bovine |
| 83 | Okada | R4 | *C. freundii* | Feaces | Bovine |
| 84 | Okada | R34 | *E. coli* | Feaces | Bovine |
| 85 | Okada | R37 | *E. coli* | Feaces | Bovine |
| 86 | Okada | R38 | *C. freundii* | Feaces | Bovine |
| 87 | Okada | R30 | *E. coli* | Feaces | Bovine |
| 88 | Benin | AB41 | *P. vulgaris* | Feaces | Bovine |
| 89 | Okada | R1 | *E. cloacae* | Feaces | Bovine |
| 90 | Okada | R23 | *E. coli* | Feaces | Bovine |
| 91 | Okada | R28 | *C. freundii* | Feaces | Bovine |
| 92 | Okada | R16 | *E. coli* | Feaces | Bovine |
| 93 | Okada | R18 | *C. freundii* | Feaces | Bovine |
| 94 | Okada | R20 | *K. pneumoniae* | Feaces | Bovine |
| 95 | Okada | R10-2 | *C. freundii* | Feaces | Bovine |
| 96 | Okada | R6-1 | *E. cloacae* | Feaces | Bovine |
| 97 | Okada | R32 | *K. pneumoniae* | Feaces | Bovine |
| 98 | Okada | R7 | *P. rettgeri* | Feaces | Bovine |
| 99 | Okada | R10-1 | *K. pneumoniae* | Feaces | Bovine |
| 100 | Benin | AB9-1 | *E. coli* | Feaces | Bovine |
| 101 | Okada | R9-1 | *K. pneumoniae* | Feaces | Bovine |
| 102 | Okada | R44-3 | *K. pneumoniae* | Feaces | Bovine |
| 103 | Okada | R52-3 | *K. pneumoniae* | Feaces | Bovine |
| 104 | Benin | AB8-1 | *E. coli* | Feaces | Bovine |
| 105 | Okada | R25-2 | *E. cloacae* | Feaces | Bovine |
| 106 | Okada | R65-3 | *E. cloacae* | Feaces | Bovine |
| 107 | Okada | R24p | *S. marcescens* | Feaces | Bovine |
| 108 | Okada | R6-2 | *K. pneumoniae* | Feaces | Bovine |
| 109 | Okada | R12-1 | *E. cloacae* | Feaces | Bovine |

**Table 2: Animal Isolates**
