# Supplementary material for: Genetic Characterization of Antibiotic Resistant Enterobacteriaceae Isolates From Bovine Animals and the Environment in Nigeria
Source: Front Microbiol. 2022 Feb 25;13:793541. doi: 10.3389/fmicb.2022.793541 (PMC8916115; doi:10.3389/fmicb.2022.793541)
Supplement: Supplementary file 3 [file Table_3.docx]

| Number | Location | Identification | Organism | Source |
| --- | --- | --- | --- | --- |
| 1 | Benin | 2d | *C. freundii* | Refuse dump |
| 2 | Benin | 1e | *P. vulgaris* | Refuse dump |
| 3 | Okada | OB1b | *K. pneumoniae* | Refuse dump |
| 4 | Okada | OB14 | *P. vulgaris* | Refuse dump |
| 5 | Okada | OB24 | *P. vulgaris* | Refuse dump |
| 6 | Benin | 4cm | *K. pneumoniae* | Refuse dump |
| 7 | Okada | A23 | *S. marcescens* |  |
| 8 | Okada | OB31N | *P. vulgaris* | Refuse dump |
| 9 | Okada | 7c | *K. pneumoniae* | Waste water |
| 10 | Okada | OB16 | *K. pneumoniae* | Refuse dump |
| 11 | Benin | 5cs | *C. freundii* | Waste water |
| 12 | Okada | C219 | *P. vulgaris* |  |
| 13 | Okada | OB33N | *K. pneumoniae* | Refuse dump |
| 14 | Okada | 6d | *E. aerogenes* | Waste water |
| 15 | Okada | OB25N | *C. koseri* | Refuse dump |
| 16 | Okada | 6b | *E. asburiae* | Waste water |
| 17 | Benin | 5eNs | *M. morganii* | Waste water |
| 18 | Okada | 6c | *E. aerogenes* | Waste water |
| 19 | Okada | B211 | *K. pneumoniae* |  |
| 20 | Okada | 7a | *K. pneumoniae* | Waste water |
| 21 | Okada | US1 | *K . pneumoniae* | Refuse dump |
| 22 | Okada | US22b | *K. pneumoniae* | Refuse dump |
| 23 | Okada | OB23b | *K. pneumoniae* | Refuse dump |
| 24 | Okada | OB21N | *P. vulgaris* | Refuse dump |
| 25 | Okada | US21 | *K. pneumoniae* | Refuse dump |
| 26 | Okada | TS1 | *P. vulgaris* | Soil |
| 27 | Okada | 6bN | *P. vulgaris* | Waste water |
| 28 | Okada | OB13b | *K. pneumoniae* | Refuse dump |
| 29 | Okada | US31 | *K. pneumoniae* | Refuse dump |
| 30 | Benin | 4d | *P. vulgaris* | Refuse dump |
| 31 | Benin | 5a | *C. freundii* | Waste water |
| 32 | Okada | 11a | *E. cloacae* | River |
| 33 | Okada | 6a | *E. aerogenes* | Waste water |
| 34 | Okada | US32 | *K. pneumoniae* | Refuse dump |
| 35 | Benin | 3ds | *E. aerogenes* | Refuse dump |
| 36 | Okada | OB1s | *K. oxytoca* | Refuse dump |
| 37 | Benin | 4cnm | *C. freundii* | Refuse dump |
| 38 | Benin | 5cb | *K. pneumoniae* | Waste water |
| 39 | Benin | 5eNb | *K. pneumoniae* | Waste water |
| 40 | Okada | US22s | *E. cloacae* | Refuse dump |
| 41 | Benin | 3db | *K. pneumoniae* | Refuse dump |
| 42 | Okada | OB14-2 | *M. morganii* | Refuse dump |
| 43 | Okada | TS1-3 | *M. morganii* | soil |

**Table 3: Environmental isolates**
