# Supplementary material for: Genetic Characterization of Antibiotic Resistant Enterobacteriaceae Isolates From Bovine Animals and the Environment in Nigeria
Source: Front Microbiol. 2022 Feb 25;13:793541. doi: 10.3389/fmicb.2022.793541 (PMC8916115; doi:10.3389/fmicb.2022.793541)
Supplement: Supplementary file 4 [file Table_4.docx]

**Table 4a. Susceptibility testing results showing % of resistant animal isolates**

| antibiotic | *K. quasipneumoniae [n=2]* | *K. variicola [n=2]* | *E. coli*  *[n=12]* | *P. terrae*  *[n=1]* | *S. marcescens*  *[n=1]* |
| --- | --- | --- | --- | --- | --- |
| ceftazidime | - | - | - | 100%[n=1] | - |
| cefotaxime | - | - | 8%[n=1] | 100%[n=1] | - |
| amoxicillin-clavulanic acid | 50%[n=1] | 100%[n=2] | 42%[n=5] | - | - |
| Cefoxitin | 50%[n=1] | 100%[n=2] | 33%[n=4] | 100%[n=1] | 100%[n=1] |
| cefepime | - | - | - | - | - |
| aztreonam | - | - | - | 100%[n=1] | - |
| meropenem | - | - | - | - | - |
| ciprofloxacin | - | - | - | - | - |
| Amikacin | - | - | - | 100%[n=1] | - |
| chloramphenicol | - | - | - | - | - |
| piperacillin-tazobactam | - | - | - | 100%[n=1] | - |
| ertapenem | - | - | - | - | - |
| trimethoprim | 50%[n=1] | - | 75%[n=9] | 100%[n=1] | - |
| levofloxacin | - | - | - | - | - |

**Table 4b. Susceptibility testing results showing % of resistant environmental isolates**

| Antibiotic | *K. quasipneumoniae [n=1]* | *K. variicola [n=1]* | *E. quasiroggenkampii*  *[n=1]* | *P. terrae*  *[n=1]* | *P. faecis[n=2]* | *E. hormachei*  *[n=1]* | *C. koseri*  *[n=1]* |
| --- | --- | --- | --- | --- | --- | --- | --- |
| Ceftazidime | - | - | - | 100%[n=1] | - | - | 100%[n=1] |
| Cefotaxime | - | - | - | 100%[n=1] | - | - | 100%[n=1] |
| amoxicillin-clavulanic acid | 100%[n=1] | - | 100%[n=1] | - | - | - | - |
| Cefoxitin | 100%[n=1] | - | 100%[n=1] | - | - | - | 100%[n=1] |
| Cefepime | - | - | - | - | - | - | 100%[n=1] |
| Aztreonam | - | - | - | 100%[n=1] | - | - | 100%[n=1] |
| Meropenem | - | - | - | - | - | - | - |
| Ciprofloxacin | - | - | - | - | - | - | - |
| Amikacin | - | - | - | 100%[n=1] | - | - | 100%[n=1] |
| Chloramphenicol | - | 100%[n=1] | - | - | - | - | - |
| piperacillin-tazobactam | - | - | - | - | - | - | 100%[n=1] |
| Ertapenem | - | - | - | - | - | - | - |
| trimethoprim | - | 100%[n=1} | - | 100%[n=1] | 100%[n=2] | 100%[n=1] | 100%[n=1] |
| Levofloxacin | - | - | - | - | - | - | - |
