# Supplementary material for: Genetic Characterization of Antibiotic Resistant Enterobacteriaceae Isolates From Bovine Animals and the Environment in Nigeria
Source: Front Microbiol. 2022 Feb 25;13:793541. doi: 10.3389/fmicb.2022.793541 (PMC8916115; doi:10.3389/fmicb.2022.793541)
Supplement: Supplementary file 5 [file Table_5.docx]

| **Number** | **Location** | **Identification** | **Organism** | **Source** |
| --- | --- | --- | --- | --- |
| 1 | Okada | OB21 | *Proteus faecis* | Refuse dump |
| 2 | Okada | OB16 | *Klebsiella variicola* | Refuse dump |
| 3 | Okada | OB25N | *Citrobacter koseri* | Refuse dump |
| 4 | Okada | 6b | *Enterobacter quasiroggenkampii* | Waste water |
| 5 | Okada | 6bN | *Proteus terrae* | Waste water |
| 6 | Benin | 4d | *Proteus faecis* | Refuse dump |
| 7 | Benin | 5cb | *Klebsiella quasipneumoniae* | Waste water |
| 8 | Okada | US22s | *Enterobacter hormaechei* | Refuse dump |
| 9 | Benin | AB45 | *Proteus terrae* | Bovine faeces |
| 10 | Okada | A22 | *Klebsiella quasipneumoniae* | Bovine faeces |
| 11 | Benin | AB3 | *Escherichia coli* | Bovine faeces |
| 12 | Benin | AB3L | *Escherichia coli* | Bovine faeces |
| 13 | Benin | AB79 | *Escherichia coli* | Bovine faeces |
| 14 | Okada | A41 | *Escherichia coli* | Bovine faeces |
| 15 | Okada | R23 | *Escherichia coli* | Bovine faeces |
| 16 | Okada | R45DY | *Escherichia coli* | Bovine faeces |
| 17 | Okada | R46 | *Escherichia coli* | Bovine faeces |
| 18 | Okada | R63 | *Escherichia coli* | Bovine faeces |
| 19 | Okada | R64 | *Escherichia coli* | Bovine faeces |
| 20 | Okada | R40 | *Escherichia coli* | Bovine faeces |
| 21 | Okada | R68 | *Escherichia coli* | Bovine faeces |
| 22 | Okada | R65 | *Escherichia coli* | Bovine faeces |
| 23 | Okada | R24 | *Serratia marcescens* | Bovine faeces |
| 24 | Okada | R9-1 | *Klebsiella variicola* | Bovine faeces |
| 25 | Okada | R44-3 | *Klebsiella variicola* | Bovine faeces |
| 26 | Okada | R52-3 | *Klebsiella quasipneumoniae* | Bovine faeces |

**Table 5: Species identification of resistant organisms using MALDITOF-MS and ribosomal MLST**
