# Supplementary material for: Genetic Characterization of Antibiotic Resistant Enterobacteriaceae Isolates From Bovine Animals and the Environment in Nigeria
Source: Front Microbiol. 2022 Feb 25;13:793541. doi: 10.3389/fmicb.2022.793541 (PMC8916115; doi:10.3389/fmicb.2022.793541)
Supplement: Supplementary file 6 [file Table_6.docx]

| **Id** | **Org** | **CAZ** | **CTX** | **AMC** | **FOX** | **FEP** | **ATM** | **MEM** | **CIP** | **AK** | **ETP** | **TZP** | **C** | **LEV** | **W** | **Resistance pattern** | **No of antibiotics resistant to** |
| --- | --- | --- | --- | --- | --- | --- | --- | --- | --- | --- | --- | --- | --- | --- | --- | --- | --- |
| R64 | *E. coli* | 28s | 27s | 25s | 24s | 34s | 33s | 33s | 33s | 24s | 33s | 24s | 23s | 33s | r | W | 1 |
| R68 | *E. coli* | 22s | 24s | 12r | 10r | 33s | 28s | 31s | 27s | 20s | 34s | 25s | 24s | 26s | 27s | AMC,FOX | 2 |
| R65 | *E. coli* | 25s | 26s | r | r | 30s | 31s | 30s | 31s | 23s | 32s | 28s | 24s | 29s | 29s | AMC,FOX | 2 |
| R63 | *E. coli* | 23s | 28s | 21s | 25s | 29s | 30s | 29s | 31s | 21s | 30s | 24s | 24s | 34s | r | W | 1 |
| R46 | *E. coli* | 26s | 28s | 21s | 21s | 30s | 29s | 31s | 23s | 22s | 34s | 22s | 22s | 23s | r | W | 1 |
| AB79 | *E. coli* | 25s | r | 21s | 25s | 31s | 30s | 33s | 29s | 21s | 31s | 23s | 20s | 30s | r | CTX,W | 2 |
| AB3 | *E. coli* | 25s | 21s | 21s | 22s | 32s | 31s | 30s | 33s | 23s | 32s | 22s | 23s | 28s | r | W | 1 |
| A41 | *E. coli* | 26s | 29s | 22s | 27s | 31s | 30s | 33s | 32s | 24s | 34s | 24s | 24s | 34s | r | W | 1 |
| A22 | *K. quasipneumoniae* | 25s | 29s | 25s | 23s | 30s | 31s | 32s | 29s | 21s | 31s | 20s | 26s | 29s | r | W | 1 |
| AB3L | *E. coli* | 25s | 29s | 22s | 25s | 32s | 31s | 32s | 34s | 24s | 35s | 25s | 23s | 30s | r | W | 1 |
| AB45 | *P. terrae* | R | r | 21s | 17r | 23i | 12r | 23s | 27s | r | 27s | 16r | 21s | 27s | r | CAZ,CTX,FOX,ATM,AK,TZP,W | **7 MDR** |
| R40 | *E. coli* | 24s | 25s | 10r | r | 30s | 29s | 31s | 24s | 21s | 30s | 21s | 21s | 21i | r | AMC,FOX,W | **3 MDR** |
| R23 | *E. coli* | 24s | 25s | 12r | 9r | 30s | 30s | 34s | 32s | 21s | 32s | 20s | 21s | 27s | r | AMC,FOX,W | **3 MDR** |
| R45DY | *E. coli* | 25s | 28s | 18r | 23s | 32s | 31s | 33s | 32s | 22s | 33s | 20s | 22s | 30s | 26s | AMC | 1 |
| R9-1 | *K. variicola* | 25s | 26s | 15r | r | 30s | 30s | 31s | 30s | 21s | 34s | 23s | 23s | 29s | 24s | AMC,FOX | 2 |
| R44-3 | *K. variicola* | 23s | 25s | r | r | 32s | 30s | 30s | 28s | 21s | 29s | 21s | 25s | 29s | 23s | AMC,FOX | 2 |
| R52-3 | *K. quasipneumoniae* | 25s | 25s | 9r | r | 30s | 31s | 32s | 28s | 20s | 29s | 20s | 24s | 27s | 24s | AMC,FOX | 2 |
| R24 | *S. marcescens* | 26s | 24s | r** | 18r** | 31s | 28s | 32s | 32s | 24s | 33s | 27s | 23s | 31s | 23s | AMC, FOX | 2 |
| OB16* | *K. variicola* | 26s | 26s | r | r | 32s | 30s | 33s | 29s | 21s | 34s | 23s | 24s | 30s | 25s | AMC,FOX | 2 |
| 5cb* | *K. quasipneumoniae* | 25s | 24s | 20s | 21s | 27s | 31s | 29s | 29s | 22s | 32s | 23s | r | 28s | r | C,W | 2 |
| US22s* | *E. hormachei* | 24s | 25s | 11r** | r** | 30s | 32s | 34s | 30s | 21s | 30s | 24s | 20s | 30s | r | AMC, FOX, W | 3 |
| OB25N* | *C. koseri* | r | r | 22s | 16r | 20r | 11r | 23s | 26s | r | 25s | 16r | 28s | 28s | r | CAZ,CTX,FOX,FEP,ATM,AK,TZP,W | **8 MDR** |
| 6b* | *E. quasiroggenkampii* | 20i | 23s | r** | r** | 30s | 29s | 29s | 34s | 22s | 23i | 24s | 20s | 32s | 26s | AMC,FOX | 2 |
| 4d* | *P. faecis* | 30s | 33s | 25s | 29s | 34s | 38s | 35s | 30s | 24s | 35s | 30s | 21s | 29s | 14r | W | 1 |
| OB21* | *P. faecis* | 29s | 30s | 25s | 25s | 34s | 31s | 34s | 29s | 24s | 34s | 30s | 24s | 30s | 13r | W | 1 |
| 6bN* | *P. terrae* | r | r | 22s | 20s | 25s | r | 24s | 28s | r | 28s | 18i | 23s | 31s | r | CAZ,CTX,ATM,AK,W | **5 MDR** |

**carbapenems**; MEM- meropenem10µg, ETP- ertapenem 10µg, **extended spectrum cephalosporins**; CAZ-ceftazidime 10µg, CTX-cefotaxime 5µg, FEP- cefepime-30µg **, penicillin+β-lactamase inhibitor**; AMC- amoxicillin-clavulanic acid 30µg, **cephamycin**; FOX-cefoxitin 30µg , **monobactam**; ATM- aztreonam 30µg, **folate pathway inhibitors**; W-trimethoprim 5µg , **fluoroquinolones**; CIP- ciprofloxacin 5µg,LEV- levofloxacin 5µg , **aminoglycosides**; AK- amikacin 30µg, **antipseudomonal penicillins + β-lactamase inhibitor**; TZP- piperacillin-tazobactam 36µg, phenicols; C- chloramphenicol 30µg *-Environmental isolates **-Antibiotics to which isolates had intrinsic resistance where not used in evaluating multidrug resistance

**Table 6: Susceptibility test results and resistance pattern of the resistant animal and environmental isolates**
