# Supplementary material for: Genetic Characterization of Antibiotic Resistant Enterobacteriaceae Isolates From Bovine Animals and the Environment in Nigeria
Source: Front Microbiol. 2022 Feb 25;13:793541. doi: 10.3389/fmicb.2022.793541 (PMC8916115; doi:10.3389/fmicb.2022.793541)
Supplement: Supplementary file 8 [file Table_8.docx]

| Type of isolates | No of strains | % no of strains | Type of resistance | Phenotypic multidrug resistance | Antibiotic resistance genes |
| --- | --- | --- | --- | --- | --- |
| Animal | 1 | 6 | MDR | Cephalosporins:ceftazidime, cefotaxime  Cephamycins:cefoxitin  Monobactams:Aztreonam  Aminoglycosides:Amikacin  Antipseudomonal penicillins + β-lactamase inhibitor: piperacillin/tazobactam  Folate pathway inhibitors: trimethoprim | CRP |
| Animal | 2 | 11 | MDR | Cephamycins:cefoxitin  Folate pathway inhibitors: trimethoprim  penicillin+β-lactamase inhibitor: amoxicillin/clavulanic acid | *str*A, *str*B, *Kdp*E, *APH*(6)-ld, *acr*D, *bla*_TEM-1B_, *bla*_AMPH_, *bla*_AMPC1_, *Glp*T, *mdt*G, *tet*(A), *emr*K, *emr*Y, *dfr*A14, *QnrS*1, *emr*R, *emr*A, *emr*B, *mdt*H, *sul*2, *ept*A, *bac*A, *Yoj*I, PmrF,msbA,Ef-Tu mutants,*sox*R,*sox*S,*mar*R, *acr*R, CRP, *acr*A, *acr*B,*bae*R, *bae*S, *cpx*A, *mdt*A, *mdt*B, *mdt*C, *mar*A, *mdt*N,*mdt*P,*mdt*O,H-NS, *gad*X, *mdt*F, *mdt*E, *evg*A, *evg*S, *Acr*E, *Tol*C, *Acr*F, *Acr*S, *emr*E |
| Environmental | 1 | 13 | MDR | Cephalosporins: ceftazidime, cefotaxime, cefepime  Cephamycins: cefoxitin  Monobactams: Aztreonam  Aminoglycosides: Amikacin  Antipseudomonal penicillins + β-lactamase inhibitor: piperacillin/tazobactam  Folate pathway inhibitors: trimethoprim | *bla*_MAL1_, *bla*_CKO-1_, Ef-Tu mutants,CRP |
| Environmental | 1 | 13 | MDR | Cephalosporins: ceftazidime, cefotaxime  Monobactams: Aztreonam  Aminoglycosides: Amikacin  Folate pathway inhibitors: trimethoprim | CRP |

**Table 8 Correlation between phenotypic and genotypic Multi-drug resistance patterns**
